# Faith in localisation? The experiences of local faith actors engaging with the international humanitarian system in South Sudan

**DOI:** 10.1186/s41018-021-00113-8

**Published:** 2022-01-11

**Authors:** Olivia Wilkinson, Kuyang Harriet Logo, Emma Tomalin, Wani Laki Anthony, Florine De Wolf, Asha Kurien

**Affiliations:** 1 Joint Learning Initiative on Faith and Local Communities, 1220 L Street NW, Ste 100 #514, Washington, DC 20005 USA; 2 University of Juba, Juba, South Sudan; 3 University of Leeds, Leeds, UK; 4 Independent Consultant, Juba, South Sudan; 5 Independent Consultant, Brussels, Belgium; 6 Tearfund, 100 Church Rd, Teddington, UK

**Keywords:** Humanitarian, Faith, Religion, South Sudan, Localisation, Local

## Abstract

Localisation, as it aims to shift power in the humanitarian system, will involve the increased inclusion of local faith actors, those national and local faith-affiliated groups and organisations that are often first, and last, responders in crises and have been responding in humanitarian contexts for many years, but often in parallel to humanitarian coordination mechanisms. In primary research in South Sudan with local faith actors and international humanitarian actors, this article aims to examine the inroads and barriers to local faith actor involvement in the humanitarian system and the realisation of localisation with local actors such as these. The research is based on an ethnographic study in which researchers were imbedded in a humanitarian project that aimed to help bridge divides between local faith actors and the international humanitarian system. The findings are based on one-on-one and group interviews with 89 participants from a range of international and local, and faith and secular, organisations. Findings indicate that local faith actors are active in responding to crises and want to be linked to the humanitarian system, but they feel distanced from it and pigeonholed as local faith actors. Formalisation through the appropriate registration systems and then training and networking with the humanitarian system helped them build legitimacy and feel confident to participate in humanitarian coordination. International humanitarian actors can help bridge barriers by understanding and connecting with the local faith actors and challenging their own assumptions about who local faith actors are.

## Introduction

As research and debate on religion and development have progressed over the last two decades (Bompani 2019), researchers have observed a ‘turn to religion’ among international aid actors. While the last five years have seen an increase in empirical and theoretical work to better understand the complexities and nuances of this “turn to religion”, there has been growing scepticism about the reasons behind and the effectiveness of the increased interest in religion. While the terms “religion” and “faith” are often used interchangeably in these debates, we broadly use the term “religion(s)/religious” when speaking of specific religious beliefs, practices, and institutions, and “faith(s)” to speak of the way in which religion manifests in humanitarianism, e.g. faith actors and faith engagement.

For the past decade or so, critics have drawn attention to the instrumentalisation of faith actors in aid and the limited parameters of acceptability available to faith actors as they participate in this domain (Jones and Petersen 2011; Ager and Ager 2015; Wilkinson 2019). They argued that faith actors had been used by secular international aid for their assets and they had adapted to fit within boundaries defined by the aid system (Jones and Petersen 2011; Deneulin and Bano 2009).

On the whole, the international aid system has tended to engage with those faith actors that are formally registered as NGOs, who understand the global development lexicon and avoid overt expressions of faith identity. By contrast, local faith actors (LFAs), who are a diverse cohort (see typology of LFAs below), are less likely to form partnerships with international aid organisations, particularly the more ‘local’ they are, as they are less likely to have co-opted the formal language of the international humanitarian system, including downplaying faith identities (Tomalin 2018). While these points still stand, more recent scholarship adds nuance to this by exploring the ways in which aid has never been truly “secular” (Haustein 2020; Wilkinson 2019), the influence of neo-colonial and Orientalising views of religions from development actors (Khalaf-Elledge 2020), and the influence of faith actors in playing different roles and shaping the boundaries between secular and religious spaces in aid (Tomalin 2020).

Yet much of this work still speaks of “aid,” in general, and with a predominant focus on development efforts, not humanitarian action. Humanitarian action, as actions that aim to save lives, alleviate suffering, and maintain life with dignity, orients around shorter-term needs in emergency settings. While recent efforts to see links across the triple nexus of humanitarian-development-peace work acknowledge that humanitarian action should not exist in a silo (ICVA 2018), the reality remains that international humanitarian actors have their own structures (the cluster system), norms, and standards (humanitarian principles, Core Humanitarian Standards) that distinguish this work from development and peacebuilding.

Regarding humanitarian action specifically, the literature on the role of faith actors is sparser. Ideas have paralleled some of the work in development studies, underlining that religious beliefs and practices have also been privatised, marginalised, and instrumentalised in the humanitarian system (Ager and Ager 2015). As with development, neither is there a clear point at which the humanitarian system was religious and became secular or vice versa; it is instead a story of constant and ever-changing dynamics between religious and secular positions (Wilkinson 2019). Nevertheless, recent research has shown that, just as religious values have influenced humanitarian positions over the years, so too have secular values. International humanitarians are not widely aware that their secular values influence their views and positionality (Wilkinson 2017a).

Barriers to faith engagement in the humanitarian system remain, therefore, such as international humanitarian actors’ perceptions about the abilities of LFAs to uphold the humanitarian principles, even when such concerns are not evidenced. In addition to this, LFAs share the same problem as local actors in general, often being too far removed from the international humanitarian system to understand and embody its terminology and requirements. This can result in LFAs not sufficiently bracketing their faith identity in humanitarian action (as is the norm in the formal humanitarian system) and in not aligning with donor expectations around reporting and accountability (Wilkinson and Ager 2017).

Recently, the international humanitarian system has committed to an intentional reorientation through strengthening and enabling the role of local humanitarian actors. One of the key initiatives in this policy arena is the ‘Grand Bargain’ commitments made at the World Humanitarian Summit in 2016, which aimed to achieve the goal of providing access to at least 25% of international humanitarian funding “as directly as possible” to local and national actors by 2020 (World Humanitarian Summit 2016, 5). Among other things, the Grand Bargain encourages humanitarian actors to “understand better and work to remove or reduce barriers that prevent organisations and donors from partnering with local and national responders…” (World Humanitarian Summit 2016, 5) and increase the participation of local communities in humanitarian decision making (World Humanitarian Summit 2016, 10). Against the back-drop of the pre-existing ‘turn to religion’ within international aid, and given the centrality of religion and LFAs in the lives of local communities in the global south facing humanitarian crises, we might expect to see LFAs take on a new level of visibility and influence in the humanitarian sector. However, this has not been the case. Drawing upon research carried out in South Sudan between 2018 and 2019, we examine the experiences of LFAs engaging with the international humanitarian system and seek to better understand the barriers to *faith in localisation.*

The terms (indeed those that we use throughout this article, as the necessary although incomplete shorthand) “international humanitarian system” and “local faith actors” set up binaries that are not, in fact, clearly delineated. The first binary is between the local and the international, the second between faith-based and humanitarian (inferred as the difference between religious and secular backgrounds). We need to examine the interaction between these categories to understand the full experience of local faith actors in humanitarian action—most importantly, LFAs should not be essentialised as local/national NGOs, nor only as faith actors, since they represent both categories and the interplay between them. Their position in relation to the humanitarian system is impacted by both designations. Power is delineated among the lines of both local/international and faith/secular divides, resulting in the reality that local faith actors can be doubly marginalised (alongside and adding to other marginalisation they may experience based on gender, race, age, ethnicity, etc.).

Drawing upon existing literature, we begin this paper with a critical examination of “localisation” discourses, which reveals the power dynamics of international-local divides in the humanitarian system and the limits of localisation efforts as they currently stand to effectively address these. This is followed by a discussion of the relationship between LFAs and localisation, including looking at LFAs as local humanitarian responders as well as the reasons why the international aid system has an uneasy relationship with them. Then we turn to our findings from research with LFAs and humanitarian actors in South Sudan. Here we bring forward perspectives from LFAs and humanitarian actors in South Sudan against the backdrop of an innovative project called Bridging the Gap (BtG), funded by the Belgian Government and implemented by a consortium of partners (Tearfund, Tearfund Belgium, RedR UK, Islamic Relief Worldwide, Islamic Relief in South Sudan, the Joint Learning Initiative on Faith and Local Communities [JLI], and the University of Leeds). The project aimed to address some of the issues mentioned above by training LFAs in humanitarian processes and standards and providing networking opportunities between LFAs and international humanitarian actors so that the LFAs could establish partnership possibilities and international humanitarian actors could understand and learn from the work of LFAs. We suggest that capacity sharing exercises, such as BtG, in which LFAs can learn about humanitarian standards and humanitarian actors can learn from LFAs, are particularly needed for localisation to be sustained and effective in the humanitarian system.

## The limits to “localisation”

The localisation approaches taken by some INGOs and donors have been critiqued as a form of remote management taken by stakeholders who desire to control funding and projects without the security risks and logistics needed to start significant operations in a country. Dixon et al. (2016), in their research on Syrian NGOs, outline the difference between remote management and inclusive partnership. Remote management instrumentalises local NGOs to access locations and achieve INGO-defined aims, while inclusive partnerships seek inputs in decision making from all actors (local and international) involved. These inclusive partnerships “are characterised by pragmatism and flexibility. The language and actions employed by partners reflect a view that local actors are more than just a vehicle for access. Working with local actors is not seen simply as a means to an end, but an end in itself” (Dixon et al. 2016).

Funding arrangements are a primary impediment to localisation. The requirements on local actors to have audited records, elaborate financial systems, policies, and reporting procedures in place before they are funded becomes a barrier and a vicious cycle: without funding, local actors cannot institute these systems, but without these systems, they cannot cross eligibility thresholds to gain funding. While these requirements are often well-intentioned (targeting corruption, protecting whistle-blowers, etc.), processes can be needlessly complicated and prohibitive for local actors with relatively small budgets. As of 2019, some progress had been made with, for example 39% of South Sudan’s UN Country Based Pooled Funds going to national NGOs (Charter 4 Change 2020a). Yet even as researchers debated the impact of the COVID-19 pandemic for localisation in humanitarian response (with travel restrictions in place, has the pandemic revealed that overseas aid and the era of the expat cannot continue?) (Aly 2020), the localisation advocacy coalition, Charter4Change, found that only 0.1% of official COVID-19 humanitarian funding had been directed to local NGOs by May 2020 (Charter for Change 2020b), a long way from the 25% goal of the Grand Bargain.

These funding impediments are also linked to conversations around “capacity.” Capacity—what it is and who has it—is often defined by the funders and international actors. As Fast and Bennet (2020, 17) put it, “Organisations that possess capacity determine how to prioritise skills and abilities and, in turn, determine which organisations have the “right” kind. The same organisations usually assume that capacity, capability and expertise flow in a single direction: from international to national organisations.” Local actors are often not given the space to define their capacities but are required to meet pre-determined capacities as set by international actors.

Recent debate has re-framed this as a question of capacities versus complementarities, asking if it is more appropriate to find what complementarities exist between local and international organisations (e.g. an international organisation has financial management capacity and a local organisations complements this with community knowledge and access) (Barbelet 2019). Organisations defining these capacities are highly risk averse and assert regular due diligence checks to monitor local partners who have strived to reach these pre-determined capacities. One local actor, from the NGO Centre for Community Health and Development International (CHAD) in Nigeria, implored international actors to change their ways:

These endless due diligence checks (we have had three in the last 12 months alone) continue to portray us and other NGOs like us in a negative light, throwing the spotlight on our limited capacity as a justification for why funds should go to the international NGOs instead… We urge you to be a little bit more trusting and a little bit less fixed in your approach to engaging effectively with local actors. A little bit smarter, a little less punitive of our inadequacies and a lot more attentive to what genuine and hardworking local actors like CHAD actually need (Usen 2019, 79).

As this author states in the title of the piece, local actors are “frustrated, not stupid!” Ultimately this leads to an unjust situation that Fiddian-Qasmiyeh has called the paradox of localisation: “[localisation] aims to ‘support’ specific ‘local’ (read ‘Southern state and regional’) responses precisely by institutionalising them within the broader paradigm and parameters established by the “international system”, even when that international system has previously actively dismissed and mistrusted those actors” (Fiddian-Qasmiyeh 2018b). Local actors are invited into the humanitarian system but told within almost the same breath that they are not sufficient.

Yet we must still explore the basic supposition that there is a “local” and “international”. What is “the local”? As has been stated by others, the local is not homogenous (Robillard et al. 2020) and represents an incredibly large range of actors. Some have stated that it is even wrong to construct a local/international binary, as it diminishes the power dynamics and hierarchies that are also at play within “the local” time and space: “Constructing them as binary opposites is problematic as it risks reproducing stereotypes and current power asymmetries within the humanitarian system through a focus on Western international actors and a preference towards dominant local or non-Western international elites. In contrast, a critical reading of the local allows capturing the complex dynamics of intervention processes and the trans-local and transcultural entangled relationships of humanitarian actors within particular humanitarian contexts” (Roepstorff 2020, 292). Other conversations exist around ideas of the global North and South (also contested terms) and the importance of shifting away from the ideas of global north knowledge, capacity, and finances going in one direction towards the global south. The focus should rather be placed on the fact that South-South cooperation and local solidarity mechanisms constitute the majority of assistance provided towards people living through humanitarian crises, and on finding ways to counter inequalities in the international humanitarian system (Fiddian-Qasmiyeh 2018a). If external aid can be as disruptive to local cultures as it is helpful (Binder and Baker 2017), we should instead be thinking of modes of mutual aid and cooperation that leave international assistance if not out of the equation, at least a complementary part of it.

## Localisation and local faith actors

The limits of localisation hindered the possibility of enhancing LFAs roles in humanitarian action, even given the “turn to religion” in aid over the past couple of decades. This is despite the fact that local faith actors have many qualities that are critical to enable timely and effective humanitarian support to communities. In research on the role of local faith actors in the 2014-2015 Ebola response, it was highlighted that their assets include their values/motivation, access to remote locations and communities, trusted position among community members, long-term presence, and knowledge of communities (Featherstone 2015). Other reports add to this to include their authority, their resources (financial, infrastructural, personnel), presence as first *and* last responders, and presence across all sectors of humanitarian response (Wilkinson 2017b, 8–9). In South Sudan, previous research has shown that LFAs particularly act as first responders (hosting tens of thousands in church compounds when they are displaced, for example) (Glinski 2017) and as mediators (negotiating access for distributions, for example) (Tanner and Moro 2016). Yet it is not simply the positive assets that must be highlighted but also the complexity of LFAs, where they can present different faces to different donors (Fiddian-Qasmiyeh 2011) and need to be understood in their context to explore how processes of inclusion and exclusion foreground some LFAs over others and how their faith identity intersects with other identities (Carpi 2018; Tadros and Sabates-Wheeler 2020; Wilkinson and Eggert 2021).

Where there is engagement, instrumentalisation is an issue that affects LFAs due to their advantageous qualities. Ager and Ager explain that “The focus is on the physical and social resources of faith communities… This emphasis is evident in the vocabulary that is now frequently adopted to justify humanitarian engagement with religion: religious communities have important ‘resources’, ‘tools’, or ‘outreach capacities’….” (Ager and Ager 2015, 64-65). They note that such partnerships “undermine the legitimacy and authority of the reasoning and reflection of people of faith in humanitarian contexts” and make for “highly conditional” engagements (Ager and Ager 2015, 64-65), in which the power is very clearly held by the external, international actor and not the local faith actor. International humanitarian actors should start by being careful with their language - partnerships are not about “using” or “leveraging” local assets, but finding ways to equitably partner with and support local capacities.

Local faith actors are often part of international religious networks that provide their own funding sources and have helped build capacity over many decades. For example, Kinney notes that the Episcopal Church in South Sudan has had a formal relationship with the Church of England’s Salisbury diocese since 1972 (Kinney 2012, 753). Many international FBOs are “localised” already because they have these natural links through religious structures to local faith communities. Other humanitarian organisations may perceive that they do not need to partner with and build capacity with LFAs because the international FBOs and other transnational religious networks are already partnering with them and funding them.

LFAs can be involved in parallel coordination structures that operate without communication with the humanitarian system. LFAs have their own, often highly complex, structures such as the provinces and dioceses of different Christian denominations in South Sudan. This means that there is already a high level of coordination required to manage relationships between people in the structure. While this structure can be a strong advantage for LFAs in humanitarian response because they already have a system through which to reach hard-to-access regions, it also means that it can be difficult for others to understand and navigate this structure and difficult for those within the structure to find time and space to coordinate with the equally complicated humanitarian system. In research on local faith actors and localisation in refugee response, one interviewee from an international humanitarian organisation explained:

Local faith [actors] feel unconfident about entering that arena [humanitarian clusters] ...they don’t use the same terminology...aid professionals have in many ways made themselves quite exclusive...most people don’t know what [humanitarian terminology] means! Part of it is reluctance because of a lack of confidence...and also, they are not confident that joining clusters will benefit them because they perceive a prejudice in terms of where funding will go...they think it will go to secular agencies. To some extent that is true. A lot of funders have been reluctant to work with faith based partners (Wilkinson and Ager 2017, 44).

In this way, the hierarchy of the humanitarian system has worked to exclude many LFAs, not only through a lack of understanding about how and why to include them, but also internal biases about with whom to engage. Faith actors come from their own religious systems that are not well understood by humanitarians and humanitarians have a system that is not clearly understood by faith actors, leading to points of tension and misunderstanding.

Humanitarian actors are also prone to identifying only the most obvious local faith actors, often sitting at the national capital level and comprised of high-level religious leaders—who are sometimes not as representative of the population as they portray or do not have the impact that the humanitarian actors require (Gingerich et al. 2017, 7). To further elucidate the types of local faith actors that might be involved in these debates, we defined a typology of local faith actors for the project in South Sudan, which we report on in this article (Wilkinson et al. 2020, 8):

- “Formal faith actors and networks, such as interreligious councils, that have a national or regional reach, are frequently partners with government ministries, and are generally located in the national capital. They may also have links to the UN and other international processes, including through their participation in worldwide religious networks.
- Smaller formal faith actors, which have some transnational ties, but are not linked to the UN or international development organisations. They may be supported by a few religious centres in the West (churches, mosques, etc.) but with no further international ties.
- Informal faith actors carrying out development and humanitarian work, which are small-scale and local, may be linked to local places of worship. This could include parish committees or zakat committees. They are less likely to have formal links to the UN and other international processes. They have some organisational structure within their religious community, but they are not separate, registered organisations.
- Religious leaders who can be valuable allies in promoting humanitarian goals and the Sustainable Development Goals (SDGs). It is also, however, important to engage with them when their views might hinder advancement of these goals. Religious leaders span local, national and international levels of formal and non-formal leadership.
- Places of worship and their communities which may support development and humanitarian work but are less likely to have a formal link to the UN and other international processes. Groups may spontaneously mobilise at these places of worship and within these communities when there is a crisis.”

In recent research, the United States Institute of Peace mapped religious actors in South Sudan and found, for example, that the South Sudanese Council of Churches is well-respected and liked (Notably, the Council of Churches are a strong local player with a carefully designed Action Plan for Peace and influence at the highest levels for peace promotion. They took part in the visit of President Salva Kiir, and now Vice President Riek Machar to Rome to meet the Pope in 2019.). However, it does not have the widespread representation that some might assume (Wilson 2019). This is not a reason to dismiss partnership with them, but a reason to consider other local faith actors as well.

Research on local faith communities in refugee response has pointed to a prioritisation of Christian actors over other faith actors for partnership because of a greater familiarity on the part of Western NGOs with Christian structures and hierarchies (Wilkinson and Ager 2017, 42–43). Although Muslims are impacted by many of the largest humanitarian crises of our present day, Muslim organisations, and especially local faith-based organisations that have Muslim affiliations, are marginalised in the international system. Zaman’s (2016) work on Iraqi refugees in Damascus prior to the Syrian conflict describes this: “International humanitarian organisations, including United Nations (UN) agencies, simply do not share a common “script” with local Islamic faith-based welfare service providers.” He continues: “Instead, they find it easier to engage with churches that have transnational connections with other faith-based international NGOs. As such, church organisations are better positioned to articulate their welfare activities in a secular frame than their Muslim counterparts.”

One underlying reason is a lack of knowledge and understanding - larger humanitarian institutions do not know how to manage these relationships or even understand that these relationships are possible. A second underlying reason is that narratives on securitisation and countering violent extremism have also furthered a level of institutional bias in which networks of mosques are associated with extremism, money laundering, and other risks (El Taraboulsi-McCarthy and Cimatti 2018). This is an uncomfortable aspect to the angle faith can add to the localisation debate. As related by El Nakib and Ager (2015, 42) in Jordan, a Sheikh who was head of a local organisation said: “We have no problem in partnering with organisations. However, they would not like to partner with us. Let us not play games here. We are Islamists. They would not be looking for partnership with Islamists, would they?” The question remains as to which local actors will be deemed “acceptable”, with religious identity acting as one of the ways in which local actors might be deemed ‘unacceptable’ by the humanitarian system at large. Local actors can also use these international humanitarian preconceptions for their benefit as has been shown by Fiddian-Qasmiyeh in research on Sahrawi refugee camps: the Sahrawi refugees’ political representatives “mobilised religiously-related claims to maximise diverse short- and long-term benefits both inside and outside the camps” (Fiddian-Qasmiyeh 2011, 533).

The context of South Sudan adds layers of nuance to this debate. Religious institutions have played a significant role in peacebuilding over the years, interfacing and mediating with other local, national, regional, and international governance structures invested in peace (Agensky 2019). While conflicting parties in the country are not currently divided on religious lines, Sudan is predominantly Muslim and South Sudan is largely Christian. While people in South Sudan experienced years as the minority religion as part of Sudan, the creation of South Sudan has brought Christians into the majority and Muslims into the minority (now estimated around 10%, although there has not been a recent census to confirm this).

Religion has a legitimising power (van Meerkerk and Bartelink 2015) which can be used to support social development or further entrench divides. There are many ways in which faith-based and non-faith-based actors in the humanitarian system have continued structures of inequality. Usual criticisms of faith-based actors include that they are biased towards their co-religionists (lack impartiality), unprofessional, and even dangerous in the actions they take for proselytisation. These have been valid in certain circumstances. Examples range from a group proselytising in Afghanistan that led to the killings of another group who were mistaken for them (Davies 2010), aid tied to conversion following the tsunami in Sri Lanka (Hertzberg 2015), and the large numbers of evangelical organisations arriving in Haiti following the earthquake in 2010 and resulting in the high-profile case of missionaries charged with kidnapping children (Quinn 2010).

However, there “are many small, local religious actors, from local religious leaders to relatively large national faith-based organisations, who will see that providing assistance is part of their faith and that discussion of their faith with the people they are helping is an inherent part of the whole initiative” (Wilkinson and Ager 2017, 48). We should not underestimate the agency of local actors and crisis-affected communities to engage in complex relationships involving religious difference and respect, while also affirming that it is necessary to ensure aid is not tied to forced conversion or targeted discrimination.

The rest of this article presents our methodology to consider the question of local faith actors in localisation in South Sudan, findings from original research in partnership with local faith actors in South Sudan and analysis of the findings.

## Methodology

The research took place over the course of the Bridging the Gap (BtG) project between October 2018 and December 2019. The project aimed to address some of the issues mentioned above by providing capacity-sharing opportunities between LFAs and international humanitarian actors.

The research team was made up of researchers based in South Sudan, the UK, Belgium, and the USA. The research design was reviewed and given ethical approval by the University of Leeds, with participants signing a consent form prior to interviews. Interviews were mostly conducted in English with some in local languages (i.e. Nuer, Dinka, and Juba Arabic), then being transcribed and translated by the South Sudanese members of the research team, Kuyang Logo and Wani Laki Anthony. The quotations that we include in our discussion below have been lightly edited for readability. Interviews were conducted in a variety of locations, including the offices of organisations as well as during gaps in the BtG training sessions, which were held in local hotels. Most interviews were carried out by Logo and Wani Laki, with Wilkinson and Tomalin also participating in some during visits to South Sudan.

In addition to interviews, the design of the research follows ethnographic principles of embedded researchers observing and discussing the processes they saw around them. The South Sudanese researchers embedded themselves in the BtG project by attending coordination meetings and the training sessions that were part of the project implementation (conducted in English), as well as working from the Tearfund offices in Juba where the project was based. Given the focus of the BtG project upon challenging the balance of power, the gender composition of the South Sudan research team was also important. With one female and one male researcher, we stood a better chance of accessing perspectives that might have been otherwise shut off to researchers of the opposite sex to the research participants. The researchers had a privileged position as “insiders” on the project meaning that we could explore and observe aspects of the work that would otherwise be unseen by an external researcher. While being an “insider” can also bring challenges, such as making assumptions about what is commonly held knowledge, we aimed to avoid this by collectively reviewing transcripts of interviews and field work notes and ensuring that any gaps in detail were addressed at an early stage.

As an ethnographic study, observation and the complementary qualitative research method of semi-structured interviewing were the primary methods used. The majority of the interviews took place between February and November 2019 when the research team had fully come together and implemented the research design.

The sample intentionally sought to include the local faith actors involved in the project, the international FBO staff working on the project and staff from international humanitarian organisations and local government and South Sudanese organisations who were not involved in the project activities. The sample snowballed outwards to include connections that had perspectives on localisation of humanitarian action in South Sudan and/or knowledge of faith dynamics in South Sudan. We conducted 48 interviews of which some were group interviews with 2-5 participants, meaning there were 89 research participants in total. There were 10 interviews with consortium members, 19 with LFAs involved in the BtG project, 3 with South Sudanese institutions, 3 with secular international NGOs, 2 local/national NGOs, 2 local/national FBOs not otherwise involved in the project, and 7 international FBOs. The interview data was analysed with the computer-assisted qualitative data analysis software, Dedoose.

The composition of the research team itself reflected “international” and “local” perspectives, if reductively categorised as such. However, the reality of the research team was that each member brought particular research expertise, from monitoring and evaluation experience in NGOs, to connection with South Sudanese research and academia, knowledge of global religion and development and global humanitarian research, and linguistic and contextual skills to conduct interviews in insightful and appropriate ways. Each team member was able to use their connections among humanitarian organisations, in localisation fora, and in academia to extend the reach of the research. Reflecting on our positionality, it is often the case that research partnerships are unbalanced in humanitarian research and perspectives on localisation are starting to shine a light on this situation (Fast 2019). While this research project still does not go far enough to balance all power dynamics (with the acknowledgment that the research leads were “international”, and the research implementers were South Sudanese), the research process was a team effort, even as each team member had their particular tasks to work on.

As with any research, there were some limitations. We were not able to reach all interviewees we had hoped in the time allotted and more interviews with an even greater range of international and local organisations would have added to the depth of the data. Another limitation was connectivity with the research team who were located across three continents. Internet connectivity in South Sudan was a difficulty when using the data analysis software that relied on a steady internet connection. The analysis therefore was mostly undertaken by Florine De Wolf in Belgium. Working “embedded” with a humanitarian project was also an unusual and interesting experience, not without some difficulties, not least different management structures (the research team was employed separately from the project staff, for example) and the different paces of the research and the project. The research team aimed to feedback as often as possible to the project team, but the reality of ethnographic methods is that they move more slowly than humanitarian projects and the final analysis of the data was not ready until the very end of the project. Several delays in the project timeline also meant shifts in the research timeline were needed and that further delayed the research.

## Results

Associated documentation outlines various details of the BtG project (Wilkinson et al. 2020). The results we bring forward here are from interviewees’ perspectives that speak more generally to the questions of humanitarian localisation and the place of LFAs in it.

### LFAs have an invaluable role to play in humanitarian action

As with other research showing the involvement of LFAs in humanitarian and development work (Wilkinson et al. 2019; Greatrick et al. 2018; El Nakib and Ager 2015), the LFAs involved in this project confirmed their ability to work with local communities and their commitment to their communities. An interviewee from an LFA explained how they were the only humanitarian actors reaching a certain population:

When our people are displaced the government could not allow access to the rebel-controlled areas. But people had lived without food and we were the only ones who had the courage to give food. Where no other organisation had the courage and, if we were to sit until the other organisations had to come, our people could not have been rescued.

Another interviewee from an LFA opined,

…even though the political situation makes it very difficult for humanitarian agencies to access the area, the Bishop as a neutral person, is able to provide access for field staff to access the area.

In another area, a different interviewee from an LFA also affirmed that,

(We) need local faith actors and national NGOs to be involved in this localisation because these are the people that are related to the community, people trust them and also these are the people that can go further than the international NGOs cannot go, because of far distance, because of security situation… Again, these international NGOs sometimes, when there is a disaster or anything like instability, like the war, always they used to evacuate but local national NGOs… they’ve always been there…

Local actors in general have a sustained presence in the context, while that is not guaranteed from INGOs. Specifically, with LFAs, trusted religious leaders, such as the bishop mentioned in the quotation, allow for the negotiation of humanitarian access. Many respondents indicated that secular humanitarian response is guided by the international humanitarian standards, while LFAs are guided by both the core humanitarian standards *and* religious values. While some of those interviewed did not see any major differences in the ways both secular and religious agencies approach their work, there were some respondents who asserted that religions organisations are better because they are also guided by religious values.

One interviewee summed up this motivation in relation to faith:

It is an element of sacrificing for their people because... they are prompted, because of their faith they have in God, that you must go and serve your people in good times or bad times. That is what is unique in the faith based, local faith actors in terms of humanitarian services.

This was also reflected in interviews with those outside the project, such one interviewee from an international FBO in South Sudan who said,

many of the partners are part of the church or fall under part of the church’s structure, so I think it’s the relationship they have with the community., That’s the key one for South Sudan, so because they are part of the church, they have a respected position and maybe they’re respected by people of varying different communities…

The invaluable role that local faith actors play in South Sudan was recognised by everyone we interviewed, including secular international humanitarian actors like UN agencies. However, as the next section outlines, many of the LFAs felt like outsiders to the humanitarian system for a number of reasons.

### Insider outsiders: LFAs feeling distant from the humanitarian system

Confirming other research that local actors and local faith actors are often distanced from the humanitarian system (for example, Wall and Hedlund 2016; Wilkinson 2018; Barbelet 2019), the LFAs involved in this project confirmed that, before participation in BtG, they had experienced a distance and reluctance to become partners from international humanitarian actors. Only a few of the LFAs we spoke to had interacted with the mainstream humanitarian system—through invitations to meetings where they were not able to set the tone or lead and also to implement smaller grants alongside other humanitarian actors. It also emerged that some of the individuals in the LFAs had engaged with the mainstream humanitarian sector in their individual capacities while working in other secular humanitarian organisations. Most of the LFAs had only operated within religious circles to provide assistance and pastoral counselling to populations fleeing conflict. They have been the first responders in many situations for over 50 years in Sudan/South Sudan.

This disconnect with the international humanitarian system which has a huge presence in South Sudan led to feelings of resentment from some of the LFA participants involved in BtG. For example, one said,

We have not interacted a lot and this is because the INGOs do not want to give us a chance to work in the same sector as them… The INGOs think that if the national NGOs or the LFA actors are empowered, they will lose their jobs and that is why most of them are not giving us funding. We request funds from INGOs, they don’t tell us off directly, they are just not interested…

Another LFA staff member, recognising that humanitarian needs far outweigh the funds available still felt that there was a lack of openness and trust, but that this was not a consequence of an international-local divide, but a result of a secular-religious divide:

There is a huge competition for resources among the LFAs and the secular organisations. LFAs are transparent and use financial resources to solve the problems of those who are needy, but the secular organisations have resources, but can use the resources for other things. The secular organisations don’t believe in the capacities of the LFAs, they feel that the LFAs are ill equipped and they think that the LFAs are corrupt. The secular organisations also think that when we are given resources, we take for our own use.

This demonstrates the complex interplay of perceptions and reactions among the secular-religious dynamics of the humanitarian system. There is often an underlying sense of suspicion between different actors. The quotes from the two LFA staff members above, reveals the interchangeability of the international-local and secular-religious categories. Already, we see the local and faith grouping together as a group that is suspicious of and held in suspicion by the international and secular grouping.

### Gatekeepers: reasons for engaging with and distancing from LFAs

From the perspective of the international humanitarian actors, there was a difference between local/national actors and faith-based or secular/other civil society actors. A full range of the merits of LFA engagement in the humanitarian system were articulated by some of the international secular humanitarian actors. For example, in one of the interviews, a respondent made three critical observations: (1) that local organisations including LFAs have easy access to populations in crisis when compared with the international organisations, because they are from the local area and can easily negotiate access with any political group, in which is critical in the case of South Sudan where access is controlled by multiple political groups; (2) local organisations, including faith based ones, are part of the affected communities, making interactions, communications, and the articulation of needs and challenges, plus the delivery of assistance, much easier; (3) local organisations have a long history of amplifying and being the voices of the communities needing assistance.

There was a paradox with respect to the ways in which international humanitarian actors would recognise the importance of LFAs, but in the same breath, they would question or wonder about the feasibility of partnership with them. An individual from a secular international NGO involved in humanitarian response, but not directly involved in BtG, confirmed some of these existing divides. For example,

What has not been captured very well in the records of humanitarian work [is] how much [LFAs] are supporting humanitarian response, how much the churches are safe havens for people, how much their own initiatives are contributing into the community where the churches contribute their own food, their own clothing… [But] they are set up as church-based, [they] may not easily make them fit into the mainstream of competitiveness in the development work… looking at the number of national NGOs visiting my office each day, I never saw a faith-based organisation coming to us for a partnership, you see?

LFAs are recognised from a distance as first responders in their communities but, unless they are knocking on the doors of INGOs to ask for inclusion, they are not acknowledged as part of the “mainstream” humanitarian response. Another international humanitarian actor expressed that non-alignment with the humanitarian principles was a problem area:

…in terms of selecting our partnerships… they have to indicate their understanding of the humanitarian principles and their willingness to act in accordance with the humanitarian principles… […] maybe that’s one of the issues of capacity building more generally...

These international humanitarian actors recognise the barriers (e.g. understanding of the humanitarian principles) but also wonder why they have not been contacted by LFAs. There was no further reflection on how to reach out to LFAs or whether the impetus should be on international humanitarian actors to proactively contact LFAs, which demonstrates that the barriers to LFA participation, while recognised, are also at least unconsciously maintained. Although the BtG project also provided two training sessions for international humanitarian actors to help overcome these barriers, they were comparatively less well attended than the trainings for LFAs. This speaks to the politics of partnership where acknowledgement and understanding of the work that LFAs regularly undertake are underestimated in comparison to perceptions of LFAs around the principles.

### Overall effects of feeling of distance and actual distancing

These barriers had real and immediate effects on LFAs that were regularly reported to the interviewers. LFAs had many reflections on these barriers. For example, they expressed that retaining competent staff was difficult since the salaries paid by local faith actors are less than those paid by INGOs. Often the local NGOs train people up and then they leave for better paid jobs in INGOs. Interviewees all identified recruitment and maintaining staff as a challenge for LFAs. One example in the words of an interviewee from an LFA;

Getting funding is hard because when we write to an international organisation, for 40,000 USD for instance, we will not be given the money because we have few human resources to manage the grant. When we write project proposals, we request the services of a consultant, but consultants are also very expensive.

They are stuck in a vicious cycle of not holding the right “capacity” to receive grants and not being able to fundraise to build this capacity. While this problem is generic to all local actors, one LFA particularly spoke about the need for some of the churches to professionalise in their humanitarian staffing due to donor demands:

…an example I’d give is for educat[ing] the dioceses… they put a theologian who has not known something in finance, a theologian is doing a programme… I used to tell them, “When I come, I want to see you as project officers, I'm not coming to see you as bishops, as reverends”, because at the end of it, I have to account to a donor and therefore, “I have to treat you as a project officer, that is what it is”. So, they really need to be paid and then be told that when it comes, if it’s an accountant, put a professional accountant there.

This entirely different management and professional style required by international humanitarian donors means that LFAs are at times ill-equipped to respond to humanitarian requests in partnership with international humanitarian actors. There are tensions between the requirements to professionalise staff, the balance of working with the advantages of LFAs in their access to volunteers and the disadvantages of losing newly trained staff to INGOs. However, there were some examples given where LFAs had partnered with international humanitarian donors, as elaborated in this next section.

### Overcoming the barriers that have distanced LFAs

#### Registration

While LFAs are based in the communities where they operate and are often the first to help when there is a crisis, some of the LFAs in South Sudan are not legally registered as NGOs. Even if they are registered this might be at the local level and not meeting the legal requirement for all humanitarian actors to be registered with the Relief and Rehabilitation Commission (RRC) in Juba. Often LFAs operate informally and do not register because they lack the funds to pay for the registration, view it as a tedious process or fear the loss of independence. We also heard from some interviewees the concern that becoming too similar to secular NGOs could risk eroding the identity of LFAs, through “NGO-isation”. Others could also be lacking understanding of the need for registration and are not aware of the benefits the registration can bring. LFAs might be keener to register if they have a better understanding of what is involved and also if they are assured that they could retain their independence.

Registration was a key area of concern for the LFAs we interviewed. The LFAs all spoke widely about the advantages they saw once registered (each quote from a different LFA):

Registration is a vital first foot in the door to enable participation in the formal humanitarian system for LFAs, as one interviewee explained:

…we have been recognised. We have been registered and that is why we are able to offer services and we are able to receive such kind of training, which we were not able to get when we were not registered.

The effects of registration are then widespread such as the opportunity to receive training, the need for new bank accounts, and the internal professionalisation that promotes the visibility in the eyes of other humanitarian actors and donors, as described by this interviewee:

We registered with Islamic council and the IR [Islamic Relief] got the opportunity to meet us. Formalisation has made us more visible… We are becoming more professional. Before we were registered, we tried to apply for grants, but because we lacked the experience we failed to get the grant. The grant application process also required a bank account and we did not have one and donors did not trust us because they thought that if the money went to personal accounts it would be misused.

Registration also helps with the legitimacy of an organisation and diminish other forms of marginalisation, such as that described by an interviewee from an Islamic organisation:

We have a friendly relationship with [the] government and we are free to engage in the humanitarian sector. The government trusts us because it has seen our documents of registration.

Nevertheless, as described by this same LFA, registration is not a one-time hurdle that once achieved the LFAs can put behind them. Because of their Islamic identity, this LFA must re-register with national security. There are different barriers and forms of registration required of some LFAs over others.

#### LFA access to the UN cluster system

Although some of the LFAs we interviewed had engaged with the UN cluster system prior to being part of the BtG project, for others, it opened up new linkages. In previous years the LFAs reported some interaction with UN clusters, such as the food security cluster, protection cluster and education cluster, both at national and regional levels. Again, this participation was tied to registration and the formalisation of the organisations in the eyes of the humanitarian system’s structures:

Even with registration, other international humanitarian actors interviewed recognised that local actors are under-represented and not listened to in the clusters:

Another issue is the coordination… Yes, recently we have seen the national actors being co-ordinators, being chairs of these clusters, but if you ask yourself in terms of the authority to [make] decisions, the national ones are perhaps sometimes even bypassed, and people ought to listen...

BtG made a difference to the LFAs in that they could diversify and increase their participation in clusters or start their participation if they had not been involved before:

… we introduce ourselves [to the] food security and livelihoods cluster… so now they know us as members of that cluster… because since this project [has] come and we are selected for this Bridging the Gap project, actually we went and introduced ourselves to the Ministry of Agriculture, that we are going to be part of this cluster so whatever food security and livelihoods cluster [meetings are] going on, our representative or our officer is always attending and then the activity relating to agriculture.

The LFAs also stated the need for more coordination and their recognition that coordination was valuable, and they should play a part in it:

We have attended what we call general NGOs’ coordination meeting and when we are in that, we realise the value of clusters’ meeting which is not in existence in Kajo Keji because most of them are done either in Juba… but of course, Kajo Keji is unprivileged because of the war and so all the things are done from outside but we have strongly recommended that we need this cluster meeting, which I know in the near future is going to be there.

This LFA interviewee points out the inequalities in the coordination system in itself, in that some regions are coordinated from outside of that region, which makes it more difficult for LFAs from that region to participate in coordination. In such a case, it is not feasible for an area-based LFA to travel to Juba for meetings and they are, by default, excluded from humanitarian coordination. Overall, the findings have demonstrated deep divides between LFAs and the international humanitarian system, with LFAs recognising many of their own faults and difficulties that have led to this divide but also outlining the many ways in which the international humanitarian system has operated to exclude them, intentionally and unintentionally, from participation.

## Analysis

The history of humanitarian response in South Sudan attributes some of its success and sustainability of interventions to the role of local faith actors and faith-based organisations (Agensky 2019; Wilkinson et al. 2019; Glinski 2017). Moreover, faith actors have also been instrumental in brokering peace deals at different points in Sudan/South Sudan’s history (Wilson 2019). Furthermore, in terms of negotiating access, LFAs are better placed to accomplish that when compared to the international, secular humanitarian organisations. For instance, in South Sudan where humanitarian actors have to negotiate access between two parties—the government and the opposition—this can be very difficult and this research has demonstrated that faith actors can have an advantage at times. The parties to the conflict trust local faith leaders and allow them access, making it easy for them to reach needy populations. International humanitarian actors often overlook the roles played by faith actors when they arrive and are not proactive in identifying complementarities or forging partnerships.

The extent to which secular humanitarian actors partner with LFAs is minimal. Other international humanitarian actors agree that in times of crisis, the first responders were the churches (Glinski 2017), but do not understand the extent of their engagement and the professional capacities of the LFAs. This is in line with much of the literature that reveals the relative invisibility of many LFAs in humanitarian contexts (El Nakib and Ager 2015; Wilkinson and Ager 2017; Gingerich et al. 2017; Wurtz and Wilkinson 2020). Some international organisations were more receptive of the role that LFAs play in humanitarian response and were considering a mapping exercise to locate LFAs with whom to partner. Secular organisations interviewed noted that the localisation agenda, which is one of the pillars of the BtG project, makes a case for international actors to engage with local ones, including LFAs, because local organisations are well placed to articulate local needs of communities and to reach inaccessible places. Nevertheless, it remained the case that secular, international humanitarian actors were not widely aware of the possibilities for partnership with LFAs. Even though the discourse around localisation is now more prominent, the international humanitarian system is still fraught with power imbalances as outlined in the literature on the limited progress towards localisation (Fiddian-Qasmiyeh 2018b; Roepstorff 2020; Charter 4 Change 2020a; Usen 2019).

International humanitarian actors (faith based and secular) agree that in terms of timeframes, their time in South Sudan is limited, as is their access to remote areas of crisis. Therefore, by increasing collaborations between local actors, including LFAs, international humanitarian actors address questions around sustainability of projects and facilitate progression from crisis to recovery and into development. The projects being implemented by international humanitarian actors are time and resource bound. They are usually short term and being implemented by international staff with temporary contracts. Concerns were raised that some well-placed and well-resourced organisations chose to partner with LFAs and other local organisations only to transfer risk, for example, when speaking of humanitarian negotiations for access as described by interviewees above. When the international organisations feel they are unable to access some areas where they operate due to security concerns, they tended to outsource and not support the local actors adequately in assessing and managing security risks. While international humanitarians acknowledged the need to work with LFAs, this is still within a framework that outsources efforts and risk to local actors, as critiqued as localisation via remote management through local partners (Dixon et al. 2016), rather than equitable partnerships that build the core capacities of local actors such as LFAs and balances decision making power.

LFAs regularly mentioned challenges to their participation in the humanitarian system and as part of the broader issue of limited progress towards localisation in South Sudan. In terms of capacities, the LFAs were weak in some humanitarian capacity requirements and their interventions were often not “visible”. By contrast, international organisations were often well equipped in terms of resources (financial and human resources), and make a concerted effort to ensure their assistance is visible to the media and to donors. LFAs and international actors agreed on many points—that there is a lack of trust between international actors and LFAs, that LFAs have limited access to the formal international humanitarian coordination mechanisms, that LFAs have trust and connection with communities around shared values, and that there was confidence from communities that LFAs are committed to their humanitarian work. This is in line with much of the existing commentary of the capacities and complementarities of international and local actors to work together within the humanitarian system (Barbelet 2019). This likewise demonstrates the continued barriers in the definitions of capacity and the problems around international humanitarians being the power brokers that define what is included as “capacity” (Fast and Bennett 2020), which leaves LFAs in a weakened position, despite other clear capacities.

Several international actors expressed concerns about the nature of the assistance provided by LFAs and whether it meets internationally recognised humanitarian principles and standards. The LFAs we spoke to were committed to helping all and not discriminating based on religion—a fundamental aspect of the humanitarian principles—but needed additional capacity strengthening in operationalising the principles and standards that are prevalent in the international humanitarian system. This has been found elsewhere in that LFAs are keen to maintain impartiality for their own faith-informed reasons, as well as knowledge of the humanitarian system and standards (Wurtz and Wilkinson 2020; Wilkinson and Ager 2017). While there are still LFAs who may not adhere to these principles, this research further adds to the evidence that many LFAs uphold them.

However, some LFA interviewees stated that BtG needed to be careful not to erode the identity of LFAs, through “NGO-isation”. This concern is also reflected in wider literature, where research has brought to light the tensions with the NGO-isation of local actors and the loss of local capacity and knowledge through the professionalisation towards international standards (Borchgrevink 2017; Al-Karib 2018; Wilkinson et al. 2019). While the overall impact of the humanitarian skills training offered to LFAs as part of BtG has been to upskill and professionalise the LFAs, and this reflection on NGO-isation does not mean to undercut the impact of the training, it does however underline an overall tension in humanitarian localisation.

LFAs share the humanitarian space with UN agencies and other international humanitarian actors who have disproportionately more visibility, funding, and influence. In South Sudan, international humanitarian actors are often well connected to the state and have more financial resources to provide assistance, while many LFAs have limited experience of working in the humanitarian sector and are not well connected to government institutions like the RRC and the Ministry of Humanitarian Affairs which play a key role in sharing humanitarian updates and providing access to crisis-affected communities. LFAs share the humanitarian space with organisations that have established inter-agency networks and have access to donors and are experienced in advocating for increased donor support to programmes. LFAs, by contrast, face significant challenges with regards to creating inter agency networks and approaching donors. Overall, we find that LFAs face many of the same structural challenges and barriers to localisation experienced by local actors in general (Wall and Hedlund 2016). In this sense, much of the LFA experience is similar to other local actors. However, there are also some specific areas that set LFAs apart, including secular-religious dynamics (Ager and Ager 2015; Wilkinson 2019) that make some international humanitarians suspicious of LFAs and less inclined to partner with them. The LFAs own fears came from NGO-isation and losing their faith-based links and capacities. Nevertheless, they demonstrated an eagerness to learn the parameters of the international humanitarian system and make the most of the capacity sharing offered in the Bridging the Gap project, demonstrating that many greater efforts should be made to indeed bridge this gap between international humanitarian actors and local faith actors.

## Conclusion

Following the introduction to the literature in this field outlining the parameters of debate on faith, localisation, and the humanitarian system, the article went on to summarise the methodology of the research, then presented the results and an analysis of these results. Overall, we see differentiation across local and faith categories—not as essentialised categories with strictly defined parameters, but as concepts with blurred lines wherein local faith actors negotiate their identity among various religious and secular and international and local parameters to establish their place as legitimate humanitarian actors while maintaining their religious affiliation and community links.

If international humanitarian actors are genuinely committed to effective humanitarian support reaching crisis-affected communities, they need to move away from the assumption that local faith actors do not maintain humanitarian principles and engage with LFAs to journey with them in partnership and learning. This is why capacity sharing exercises, such as BtG, in which LFAs can learn about humanitarian standards and humanitarian actors can learn from LFAs, are particularly needed for progress towards locally led humanitarian action. Localisation approaches should make a place for the contextual capacity that LFAs bring. International humanitarian actors need to be open to true capacity sharing which includes listening to and learning from local faith actors and acknowledging their own biases.

To answer the rhetorical question of this article’s title, there is little *faith in localisation*, in both senses of the phrase. We found that there is little *faith in* the commitments towards localisation among local actors and that only limited participation is enabled for local *faith* actors in the localisation movement. However, projects like BtG have started to address this imbalance. Evidence from the project demonstrates that capacity sharing with LFAs and networking between international and local humanitarian actors can start addressing the power imbalances. Partnering with LFAs is a recognition of their invaluable work that has been largely invisible in the eyes of the broader humanitarian system and furthers localisation, affirming that LFAs should be part of the localisation agenda, just as they have always been an invaluable part of the makeup of local civil society.

## Data Availability

The dataset generated and analysed during this study are not publicly available due to privacy and protection concerns of the research participants.

## Abbreviations

BtG: Bridging the Gap project
FBO: Faith-based organisation
INGO: International non-governmental organisation
JLI: Joint Learning Initiative on Faith and Local Communities
NGO: Non-governmental organisation
LFA: Local faith actor
RRC: Relief and Rehabilitation Commission
SDG: Sustainable development goals
UN: United Nations
